# Resveratrol overcomes gefitinib resistance by increasing the intracellular gefitinib concentration and triggering apoptosis, autophagy and senescence in PC9/G NSCLC cells

**DOI:** 10.1038/srep17730

**Published:** 2015-12-04

**Authors:** Yinsong Zhu, Wenjuan He, Xiujuan Gao, Bin Li, Chenghan Mei, Rong Xu, Hui Chen

**Affiliations:** 1 Department of Pharmacology, Tongji Medical College of Huazhong University of Science and Technology, Wuhan, Hubei 430030, P.R. China

## Abstract

Gefitinib (Gef) provides clinical benefits to non-small cell lung cancer (NSCLC) patients with activating EGFR mutations. However, acquired resistance (AR) is a major obstacle to effective Gef therapy. This study demonstrated that resveratrol (Res) could synergize with Gef to inhibit the proliferation of Gef-resistant NSCLC cells. The underlying mechanisms of synergism were investigated, and the results showed that cotreatment with Gef and Res could inhibit EGFR phosphorylation by increasing intracellular Gef accumulation through the impairment of Gef elimination from PC9/G cells. Consistently, CYP1A1 and ABCG2 expression were inhibited. Meanwhile, the cotreatment significantly induced cell apoptosis, autophagy, cell cycle arrest and senescence accompanied by increased expression of cleaved caspase-3, LC3B-II, p53 and p21. Further studies revealed that autophagy inhibition enhanced apoptosis and abrogated senescence while apoptosis inhibition had no notable effect on cell autophagy and senescence during cotreatment with Gef and Res. These results indicated that in addition to apoptosis, senescence promoted by autophagy contributes to the antiproliferation effect of combined Gef and Res on PC9/G cells. In conclusion, combined treatment with Gef and Res may represent a rational strategy to overcome AR in NSCLC cells.

Among all lung cancer cases, non-small cell lung cancer (NSCLC) is the predominant subgroup (85%–90%) and is associated with a high recurrence rate and increased mortality^1^. Gefitinib (Gef), as a first-generation reversible epidermal growth factor receptor tyrosine kinase inhibitor (EGFR TKI), has been confirmed to provide clinical benefits to NSCLC patients with activating EGFR mutations^2^. Nevertheless, a vast majority of the patients initially sensitive to Gef will develop acquired resistance (AR) within 6–12 months of therapy, resulting in treatment failure^3^.

The existing mechanisms linking AR to Gef in NSCLC include EGFR T790M mutation, MET amplification, HGF overexpression, phenotypic transformation and additional genetic alterations^4^. Moreover, the increase in Gef metabolism or efflux leading to decreased accumulation of intracellular Gef may also give rise to AR^5^,^6^. However, 30% of the underlying mechanisms of resistance still remain unexplained^4^.

The complexity and diversity of AR necessitate the development of combination therapies with both molecular-targeted anticancer agents and natural products. Resveratrol (Res) is a natural polyphenol compound receiving widespread attention for its potential anticancer activity^7^,^8^,^9^. In particular, Res can reverse the resistance of cancer cells to chemotherapeutic drugs, such as doxorubicin, gemcitabine, and pemetrexed^10^,^11^,^12^, by enhancing their anticancer effects and preventing their toxic effects. However, whether Res in combination with Gef could work synergistically to overcome AR in NSCLC remains unknown.

Drug efficacy depends on the intracellular disposition process of the drug and its concentration at the target site; thus, the intracellular pharmacokinetics of anticancer drugs have become of paramount importance^13^. Roberta *et al.*^5^ found a significant difference in drug metabolism between Gef-sensitive and -resistant cell lines, while Hegedüs *et al.*^6^ observed enhanced efflux of Gef by the drug transporter ABCG2 in Gef-resistant NSCLC cells. However, the exhaustive cellular pharmacokinetic mechanisms of Gef resistance and its modulation by Res in PC9/G cells have not been clearly elucidated.

The complex balance among apoptosis, autophagy and senescence represents the linchpin that determines cell survival or death^14^. Moreover, it is noteworthy that autophagy and senescence contribute to chemotherapy in apoptosis-resistant cancer cells^15^,^16^. Intriguingly, Res is able to regulate apoptosis, autophagy and senescence, resulting in cell death by activating distinct signalling pathways^17^. However, the impact of combined Gef and Res treatment on cell fate in Gef-resistant NSCLC cells remains unclear. In this study, we dissected the synergistic effects of combined Res and Gef treatment, focusing on the mechanisms of overcoming AR to Gef in NSCLC cells and the complex interplay of apoptosis, autophagy and senescence in this process.

## Results

### Sequence-dependent interactions between Gef and Res in the PC9/G cell line.

**Figure 1:**
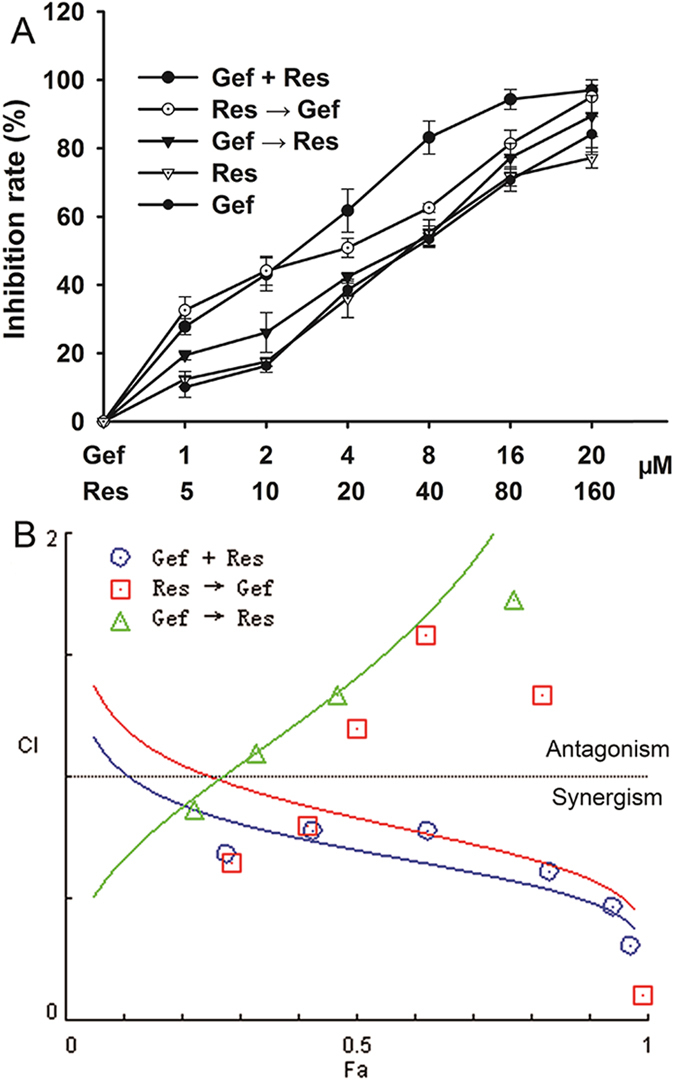
Inhibitory effects of three different treatments of Res and Gef in human NSCLC PC9/G cells. **(A)** Cells were cultured in 96-well plates and treated with the three different combination schedules for 72 h. Gef + Res: cotreatment with Gef and Res for 72 h. Res → Gef: Res pretreatment for 24 h, followed by Gef for another 48 h. Gef → Res: Gef pretreatment for 24 h, followed by Res for another 48 h. Cell viability was measured by MTT assay. Data are shown as means ± SD of three independent experiments. **(B)** The CI values of different combinations of Res and Gef were determined using the Fa-CI plot.

We successfully established a Gef-resistant NSCLC cell line PC9/G from the Gef-sensitive PC9 cell line. The IC_50_ value for Gef in PC9/G cells was 6.36 ± 1.23 μM, with a 302-fold increase relative to that in PC9 cells (0.021 ± 0.005 μM). Then, we examined the antiproliferation effects of Gef and Res in the PC9/G cell line. Both drugs inhibited cell proliferation in a dose-dependent manner (Fig. 1A).

To assess whether Res could sensitize PC9/G cells to Gef, we evaluated the effects of three different combination treatments of Gef and Res on the proliferation of PC9/G cells (Fig. 1A). Compared with Gef treatment alone (IC_50_ = 6.54 ± 0.58 μM), all three combination treatments showed decreased IC_50_ values for Gef. Apparently, the Gef + Res treatment (IC_50_ = 2.33 ± 0.27 μM) was more potent than the other two treatments (Res → Gef: IC_50_ = 2.77 ± 0.33 μM; Gef → Res: IC_50_ = 4.69 ± 0.28 μM). We then assessed the growth inhibitory effects of different drug combinations according to the combination index (CI) (Fig. 1B). As expected, the Gef + Res treatment exhibited the greatest synergistic effect (CI < 0.9) and was chosen for subsequent study.

To confirm the beneficial effect of Res in combination with Gef in other Gef-resistant NSCLC cell lines, we also evaluated the effects of the Gef and Res combination treatment on the proliferation of HCC827/G, A549, H1975, and H1299 cells. The characteristics of these NSCLC cell lines are summarized in Supplementary Table S1. The results showed that Res could induce sensitivity to Gef in all the NSCLC cell lines tested, regardless of the EGFR mutation status (Supplementary Fig. S1).

### The relationship between the intracellular Gef concentration and the inhibition rate of EGFR phosphorylation

**Figure 2:**
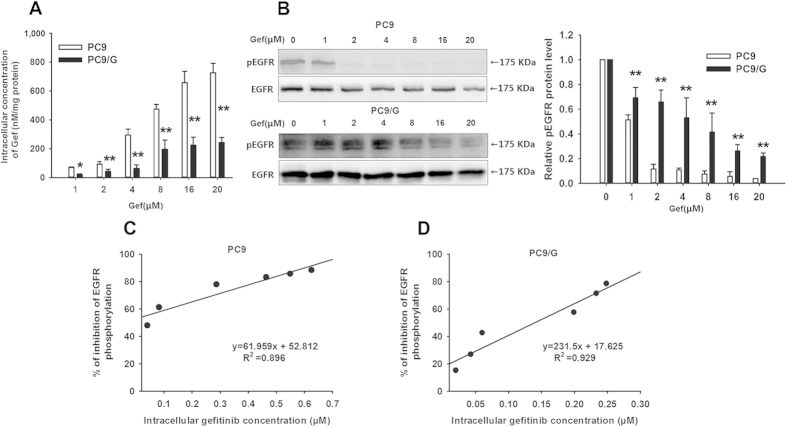
Intracellular Gef concentration in PC9 and PC9/G cell lines and its effect on EGFR phosphorylation. **(A)** Cells were incubated with the indicated extracellular Gef concentration for 8 h, and then the intracellular Gef concentration was calculated and expressed as nM/mg of protein. **(B)** Cells were incubated with the indicated extracellular Gef concentration for 8 h, and then Western blotting analysis was performed using monoclonal antibodies directed to p-EGFR (p-Tyr-1068) and EGFR. Data are presented as means ± SD (n = 3). *P < 0.05, **P < 0.01 compared with the PC9 group. **(C)** and **(D)** represent the linear correlation analysis of the relative inhibition ratio of EGFR phosphorylation and the intracellular Gef concentration in PC9 and PC9/G cells, respectively. The protein amounts at each point were quantified by densitometric analysis, and the ratios of phospho-EGFR to total EGFR were calculated. The values are expressed as the percentage of inhibition relative to the control and are plotted as a function of the intracellular Gef concentration.

As an effective EGFR TKI, Gef competes with ATP for binding at the intracellular catalytic domain of transmembrane tyrosine kinases^18^. Therefore, the intracellular Gef concentration plays a crucial role in Gef effectiveness, and EGFR phosphorylation is regarded as an appropriate marker of response to Gef therapy. When exposed to 1–20 μM Gef, the intracellular Gef concentrations of PC9/G cells were significantly lower than those of PC9 cells (P < 0.05) (Fig. 2A). Furthermore, an apparent saturation phenomenon was observed for the intracellular Gef concentration of PC9/G cells when the exposure concentration reached above 8 μM. These results indicated that the intracellular accumulation of Gef in PC9/G cells was significantly decreased due to AR. Meanwhile, at the same extracellular concentration of Gef, phosphorylated EGFR protein expression in PC9/G cells was significantly higher than in PC9 cells (P < 0.05) (Fig. 2B).

Furthermore, we found that there was a significant positive linear correlation between the inhibition rate of phosphorylated EGFR protein expression and the intracellular Gef concentration (for PC9: R^2^ = 0.896; for PC9/G: R^2^ = 0.929) (Fig. 2C,D), which confirmed that the inhibition rate of phosphorylated EGFR protein expression was dose-dependently regulated by the intracellular Gef concentration. Hence, the increase in the intracellular Gef concentration could result in an enhanced inhibitory effect on EGFR phosphorylation.

### The effect of Res on Gef intracellular pharmacokinetics and EGFR phosphorylation

**Figure 3:**
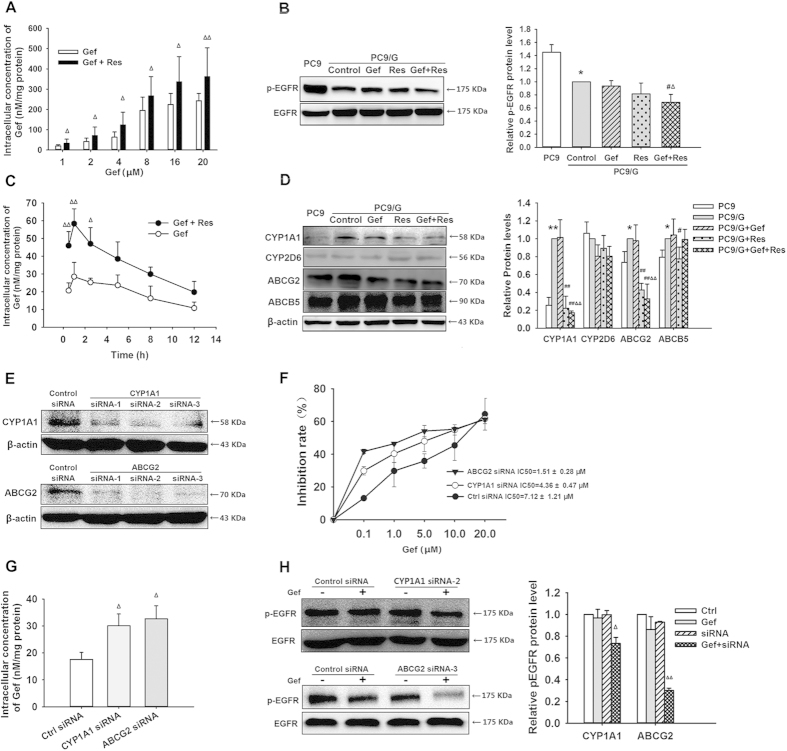
Res affected the intracellular concentration of Gef and the EGFR phosphorylation by modulating Gef-related enzymes and transporters. (**A**) PC9/G cells were incubated with the indicated concentrations of extracellular Gef alone or combined with Res (40 μM) for 8 h, and then the intracellular Gef concentration was calculated. (**B**) The expression levels of p-EGFR and EGFR were detected by Western blotting. (**C**) PC9/G cells were incubated with Gef (1 μM) alone or combined with Res (40 μM) for the indicated times, and then the intracellular Gef concentration was calculated. (**D**) The expression levels of CYP1A1, CYP2D6, ABCG2 and ABCB5 proteins were detected by Western blotting. (**E**) PC9/G cells were transfected with CYP1A1 siRNA, ABCG2 siRNA or control siRNA (80 nM) for 24 h. Western blotting was then performed to determine the protein levels of CYP1A1 and ABCG2. (**F**) Knockdown of CYP1A1 or ABCG2 increased Gef sensitivity. PC9/G cells (5 × 10^3^ cells/well) were seeded onto 96-well plates and transfected with CYP1A1 siRNA, ABCG2 siRNA, or control siRNA (80 nM) for 12 h. Then, the cells were treated with increasing concentrations of Gef for 72 h. Afterwards, cell viability was measured by MTT assay. (**G**) Knockdown of CYP1A1 or ABCG2 increased the intracellular concentration of Gef. PC9/G cells (4 × 10^5^ cells/well) were seeded in 6-well plates. At 24 h after transfection, cells were treated with 1 μM Gef for 8 h. Then, the intracellular concentrations of Gef were determined. (**H**) Knockdown of CYP1A1 or ABCG2 enhanced Gef-induced inhibition of EGFR phosphorylation. The protein expression levels of EGFR and p-EGFR were detected by Western blotting. Data are expressed as means ± SD (n = 3). *P < 0.05, **P < 0.01 compared with the PC9 group; ^#^*P* < 0.05, ^##^*P* < 0.01 compared with the PC9/G control group; ^△^*P* < 0.05, ^△△^*P* < 0.01 compared with the Gef treatment group.

PC9/G cells were incubated with Gef alone or Gef combined with Res. Then, the intracellular Gef concentrations and phosphorylated EGFR protein expression were measured. Compared with Gef alone, the Gef + Res treatment significantly increased the intracellular Gef concentration (P < 0.05) (Fig. 3A) and decreased the phosphorylated EGFR protein expression (P < 0.05) (Fig. 3B). These results demonstrated that Gef + Res treatment inhibited EGFR phosphorylation synergistically by increasing the intracellular Gef concentration.

The mean intracellular Gef concentration-time curves are shown in Fig. 3C, while the pharmacokinetic characteristics were well described by a one-compartmental model and the pharmacokinetic parameters (K_a_, K_e_, AUC, CL, T_max_, and C_max_), which is summarized in Table 1. The intracellular Gef concentrations after Gef + Res treatment were increased by an average of approximately 1.8-fold relative to those after Gef treatment alone. As expected, the clearance (CL) for Gef + Res treatment was significantly decreased by 44%, and the area under the concentration-time curve (AUC) was significantly increased by 48% compared with those for Gef treatment alone. This result suggested that Res could impair the elimination and promote the accumulation of intracellular Gef.

### Combined Gef and Res modulated the expression of Gef-related metabolism enzymes and transporters

To make a thorough inquiry into the possible mechanism for the influence of Res on intracellular Gef pharmacokinetics, the protein expression levels of CYP1A1, CYP2D6, ABCG2, and ABCB5 in PC9 and PC9/G cells were analysed (Fig. 3D). We found that PC9/G cells showed enhanced protein expression for CYP1A1, ABCG2, and ABCB5 but not for CYP2D6. Unsurprisingly, after Gef + Res treatment, CYP1A1 and ABCG2 expression decreased by 83% and 67% (P < 0.01), respectively, compared with Gef treatment alone in PC9/G cells. However, there were no significant changes in CYP2D6 and ABCB5 expression. These results indicated that Res might impede intracellular Gef metabolism and efflux by downregulating CYP1A1 and ABCG2, which partially explained the observed enhanced accumulation of intracellular Gef.

To further confirm the involvement of CYP1A1 and ABCG2 in Gef resistance, siRNAs were used to silence CYP1A1 and ABCG2 gene expression (Fig. 3E). As expected, CYP1A1 or ABCG2 knockdown resulted in enhanced proliferation inhibition in Gef-treated PC9/G cells (Fig. 3F). Moreover, CYP1A1 or ABCG2 knockdown increased the intracellular Gef concentration (Fig. 3G), as well as enhanced Gef-induced inhibition of EGFR phosphorylation (Fig. 3H). This findings further verified the significance of Gef metabolism and efflux in Gef resistance.

### Res potentiated Gef-induced apoptosis of PC9/G cells

**Figure 4:**
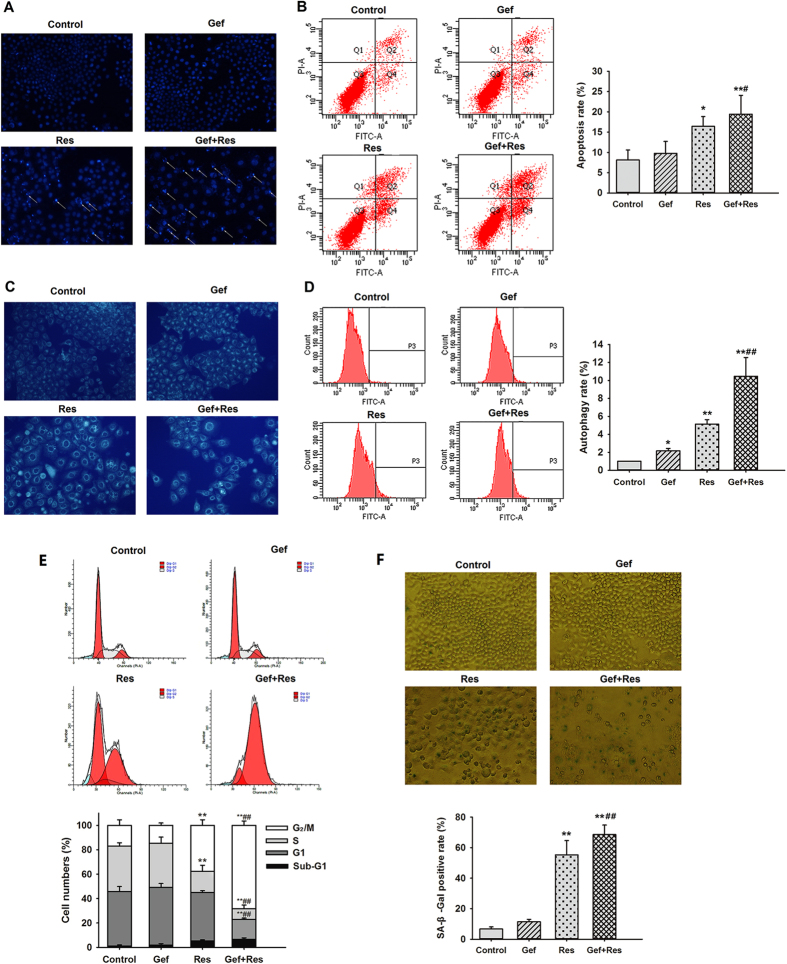
Res enhanced Gef-induced apoptosis, autophagy, G2/M phase cell cycle arrest, and senescence in PC9/G cells. Cells were treated with Gef (1 μM) alone or combined with Res (40 μM) for 72 h. **(A)** Morphological changes in PC9/G cells including nuclei condensation and fragmentation (arrow) were observed by DAPI staining under a fluorescence microscope. **(B)** Annexin V-FITC/PI staining assay of PC9/G cells was analysed by flow cytometry. **(C)** Autophagy was detected by MDC staining, and **(D)** fluorescence intensity was detected by flow cytometry. **(E)** Cells were stained with PI, and then the DNA content was analysed by flow cytometry. Sub-G1, G1, S and G2/M indicate different cell cycle phases. **(F)** Cells were stained for SA-β-Gal activity. The percentage of senescent cells in PC9/G cells was measured. Magnification: 200× . Data are presented as means ± SD (n = 3).*P < 0.05, **P < 0.01 compared with the control group; ^#^*P* < 0.05, ^##^*P* < 0.01 compared with the Gef treatment group.

**Figure 5:**
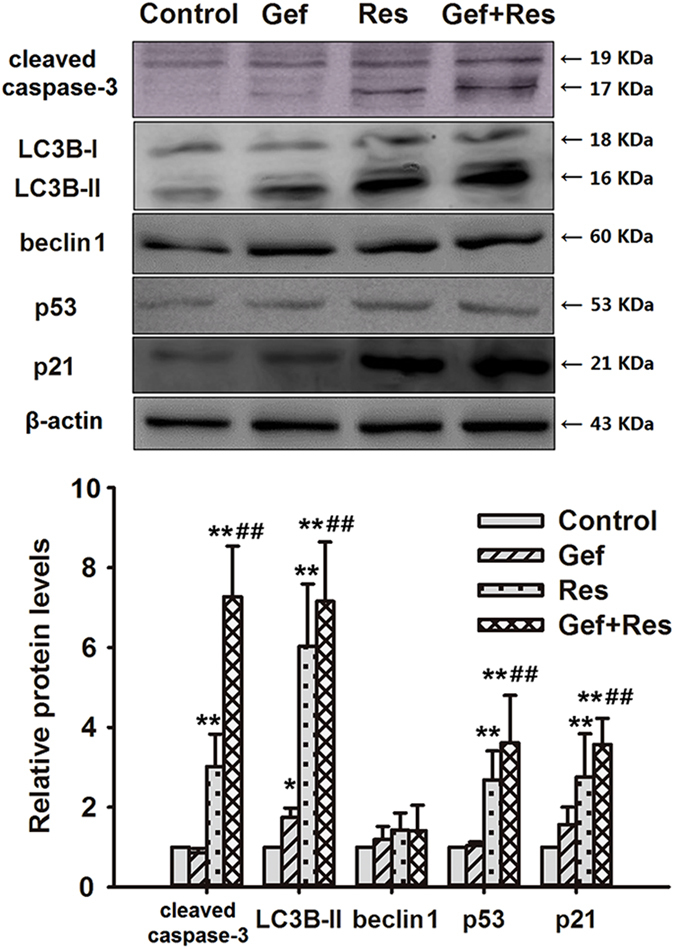
Cotreatment with Res and Gef affects the expression of apoptosis-, autophagy- and senescence-related proteins in PC9/G cells. Cells were treated with Gef (1 μM) alone or combined with Res (40 μM) for 72 h. Then, the expression levels of cleaved caspase-3, LC3B-II, beclin 1, p53, and p21 protein were analysed by Western blotting. Data are presented as means ± SD (n = 3). *P < 0.05, **P < 0.01 compared with the control group; ^#^*P* < 0.05, ^##^*P* < 0.01 compared with the Gef treatment group.

To examine whether Res could increase Gef-induced apoptosis in PC9/G cells, DAPI staining (Fig. 4A) and flow cytometric analysis (Fig. 4B) were performed. The results indicated that Gef + Res treatment notably increased the apoptosis rate in contrast to Gef treatment alone (19.43 ± 4.64 vs. 9.77 ± 2.96, P < 0.05), indicating that Res potentiated Gef-induced apoptosis. In addition, incubation with Gef + Res also led to marked cleavage of caspase-3 (Fig. 5).

### Res increased Gef-induced autophagy of PC9/G cells

As a specific *in vivo* marker of autophagic vacuoles, the MDC stain was used to assess autophagic cell death. Elevated fluorescence intensity and an increased number of MDC-labelled cells were observed in all three drug treatment groups, among which Gef + Res treatment induced the most prominent autophagy (Fig. 4C). The fluorescence intensity of MDC-labelled cells measured by flow cytometry also showed concordant results (Fig. 4D).

We further assessed two classic hallmarks of autophagy: beclin 1 expression and the conversion of LC3B I to LC3B II^19^. These results showed that there were significant increases in LC3B II protein expression in all three drug treatment groups, among which Gef + Res treatment showed the highest protein expression level of LC3B II (Fig. 5). However, no significant changes in beclin 1 protein expression were observed between the groups. Because beclin 1 is a key initiator of autophagy^20^, we speculate that beclin 1 might be upregulated during the first few hours of autophagy and then downregulated to normal levels by 72 h. A study conducted by Yunkyung Hong^20^ corroborates this hypothesis.

### Res enhanced Gef-induced G2/M phase cell cycle arrest as well as senescence of PC9/G cells

As shown in Fig. 4E, treatment with Gef + Res markedly increased the percentages of cells at the G2/M phase compared with Gef treatment alone, suggesting that Res contributed to Gef-induced cell cycle arrest at the G2/M phase. Senescence, a permanent state of cell cycle arrest, was measured using the senescence-associated beta-galactosidase (SA-β-gal) assay. As expected, obvious senescence characteristics, such as flattened and enlarged nuclei and accumulated granular particles, were observed in Gef + Res treated cells (Fig. 4F). Moreover, treatment with Gef + Res increased the proportion of SA-β-gal-positive cells compared with Gef treatment alone ((68.6 ± 6.2)% vs. (11.4 ± 1.6)%, P < 0.01).

p53 has been reported to be a critical initiator of cellular apoptosis, autophagy and senescence^21^,^22^,^23^,^24^; hence, we examined the changes in the expression of p53 and its downstream effector p21^waf1/cip1^. Our findings showed that Gef + Res treatment significantly increased p53 and p21^waf1/cip1^ expression compared with either drug alone (Fig. 5), indicating that the p53 pathway might play an important role in the cell death processes involving apoptosis, senescence and autophagy triggered by combined Gef and Res treatment.

### The interrelationship of apoptosis, autophagy, and senescence during combined Gef and Res treatment

To further investigate the complex interrelationships between apoptosis, autophagy and senescence in response to combined Gef + Res treatment, PC9/G cells were treated with the specific caspase-3 inhibitor Ac-DEVD-CHO (DEVD) (for apoptosis inhibition) or the autophagy inhibitor 3-methyladenine (3-MA) to interfere with the combination effects of Gef and Res.

**Figure 6:**
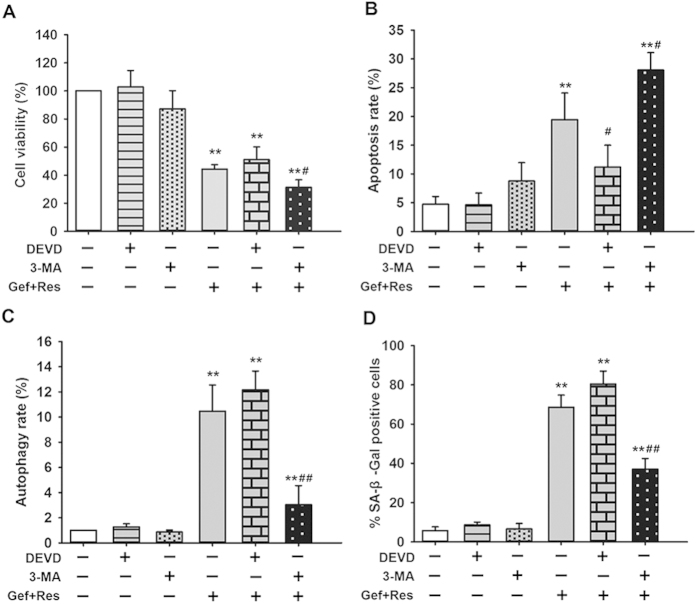
The effects of DEVD and 3-MA alone or combined with Gef + Res treatment on cell viability (**A**), apoptosis (**B**), autophagy (**C**) and senescence (**D**). PC9/G cells were treated with 3-MA (5 mM), DEVD (10 nM), and Gef (1 μM) + Res (40 μM), alone or in combination of any two, for 72 h. Then, cell viability, apoptosis, autophagy and senescence were detected as described in the Materials and methods. Data are presented as means ± SD (n = 3). ^*^P < 0.05, ^**^P < 0.01 compared with the untreated group; ^#^*P* < 0.05, ^##^*P* < 0.01 compared with the Gef + Res treated but not DEVD, and 3-MA treated group. Abbreviation: Gef: Gefitinib; Res: Resveratrol; 3-MA: 3-methyladenine; DEVD: Ac-DEVD-CHO.

Apparently, DEVD or 3-MA alone showed no toxic effects (Fig. 6A). When combined with Gef + Res treatment, DEVD had no effect on cell viability, while 3-MA significantly potentiated the reduction of cell viability (P < 0.05). This finding suggested that apoptosis might not be the principal cell death pathway involved and that autophagy may serve as a self-protective mechanism for cell survival. As expected, DEVD and 3-MA were very effective in blocking cell apoptosis (Fig. 6B) and autophagy (Fig. 6C), respectively, as triggered by combined Gef + Res treatment. DEVD had no notable effect on cell autophagy and senescence induced by combined Gef + Res treatment (Fig. 6C,D), indicating that autophagy and senescence induction were independent of apoptosis during cotreatment. Interestingly, 3-MA significantly augmented cell apoptosis (P < 0.05) and ameliorated cell senescence (P < 0.01) induced by combined Gef + Res treatment (Fig. 6B,D). This finding indicates that autophagy antagonized apoptosis and triggered senescence during cotreatment, which identifies autophagy a determinant of cell fate in controlling the balance between apoptosis and senescence.

## Discussion

Gef is recommended for use in unselected patients with NSCLC as the second- and third-line therapy after failure of first-line chemotherapy^25^. However, AR arose within 6–12 months of therapy is the major cause for Gef treatment failure in NSCLC, which finally leads to disease deterioration (local recurrence or distant metastasis) and no successful treatment is avaliable^3^. Therefore, it is necessary to identify the underlying mechanisms of AR to achieve an effective approach to overcome this resistance. Previous studies have demonstrated that Res acts as a chemosensitizer to enhance the activity of chemotherapeutic drugs by modulating one or more mechanisms of resistance^26^. In this study, we observed that cotreatment with Gef and Res exerted a synergistic antiproliferative effect on Gef-resistant NSCLC cells and that Res partially restored Gef sensitivity, which led us to further investigate the potential mechanisms for the synergistic effects of combined Gef and Res treatment.

It is well known that Gef exerts its anticancer activity at the intracellular domain of EGFR by preventing tyrosine kinase phosphorylation and subsequent activation of the downstream signalling pathway^27^. Without doubt, the intracellular pharmacokinetics of Gef play a crucial role in the estimation of Gef efficacy. Meanwhile, EGFR phosphorylation has been regarded as an appropriate marker to measure the response to Gef therapy^28^. Our study demonstrated that a decrease in the intracellular Gef concentration leads to reduced pEGFR expression, which contributes to Gef resistance in NSCLC cells. Given this finding, we examined whether Res could reverse Gef resistance in PC9/G cells by altering the intracellular pharmacokinetics of Gef. Our study demonstrated that the intracellular concentration of Gef was increased by combined Gef and Res treatment in PC9/G cells. Pharmacokinetic analysis showed significant increases in the C_max_ and AUC values, as well as a significant decrease in the CL of Gef, in the combination group. Apparently, Res increased the intracellular concentration of Gef by both impairing its elimination and promoting its accumulation, which consequently potentiated the inhibitory effect of Gef on EGFR phosphorylation and thus contributed to the amelioration of Gef resistance.

The factors that influence the cellular pharmacokinetics of drugs comprise active transport, metabolic inactivation, pH partitioning, electrochemical gradients, target binding, and other cellular activities^13^,^29^. Here, we focused on the major drug-metabolizing enzymes and transporters that are determinants of the elimination or accumulation of intracellular Gef. It was reported that Gef is mainly metabolized by CYP1A1 and CYP2D6 in NSCLC cell lines^5^. Additionally, the ABCG2 transporter is an efficient efflux pump that mediates Gef efflux, and enhanced expression of ABCG2 confers resistance to Gef 5. ABCB5, a newly discovered member of the ATP-binding cassette transporter family, has been reported to confer resistance to multiple anticancer drugs, including taxanes, anthracyclines and doxorubicin^30^,^31^. However, the relationship between ABCB5 and Gef resistance remains unclear. We found that Gef-resistant PC9/G cells exhibited higher expression levels of CYP1A1, ABCG2 and ABCB5. These trends were significantly reversed by combined Gef + Res treatment for CYP1A1 and ABCG2 but not for ABCB5. These findings indicate that AR in NSCLC cells might be related to increased metabolism mediated by CYP1A1 and enhanced efflux mediated by ABCG2 or ABCB5. The involvement of CYP1A1 and ABCG2 in Gef resistance was confirmed by increased Gef sensitivity after knockdown of CYP1A1 or ABCG2. Therefore, it is tempting to speculate that when Gef is combined with drugs capable of inhibiting CYP1A1, ABCG2 or ABCB5, there would be beneficial therapeutic effects for patients with acquired Gef resistance.

A previous review described how Res can activate apoptosis, autophagy, senescence and mitotic catastrophe leading to cell death^17^, and the anticancer effects exerted by Res are mediated by the activation of p53 and the complex p53 network^32^. p53 is a prominent tumour suppressor activated when a cell suffers cellular stress, such as DNA damage, hypoxia, heat shock and spindle damage^33^. In addition, it has been reported that p53 acts as a central node in the regulation of apoptosis, autophagy and senescence^34^,^35^,^36^. However, whether Gef + Res combination treatment in NSCLC induces multiple cell death pathways (*e.g*., apoptosis, autophagy and senescence) involving p53 requires further exploration. Our study confirmed that the Gef and Res combination treatment induced apoptosis, autophagy and senescence accompanied by elevated expression of p53 and its downstream effector p21^waf1/cip1^ in NSCLC PC9/G cells.

Our study sought to understand the interrelationship of apoptosis, autophagy and senescence following combined Gef + Res treatment. Thus, we employed DEVD (apoptosis inhibitor) and 3-MA (autophagy inhibitor) to make a further inquiry. Autophagy has been reported as a mechanism for cells to make the efficient transition from a proliferative to a senescent state^37^. Additionally, the induction of senescence is regarded as an alternative cell death modality because it leads to irreversible cell cycle arrest, which deprives cells of their proliferative ability^38^. We found that autophagy inhibition led to decreased cell viability accompanied by elevated apoptosis and reduced senescence, which supports the notion that autophagy plays as a self-protective role in cell survival by inhibiting apoptosis and triggering senescence in response to drug-induced DNA damage. Considering the fact that the inhibition of apoptosis had no notable effect on cell viability, it is reasonable to speculate that senescence, rather than apoptosis, may be the main cell death modality activated in response to DNA damage following Res plus Gef cotreatment in PC9/G cells (Fig. 6).

As EGFR TKIs, erlotinib and Gef show similar anti-tumour activity in NSCLC patients harbouring EGFR mutation^39^. A recent study found that the combination of Res and erlotinib synergistically induced cell death in NSCLC cells through apoptotic pathway mediated by PUMA and survivin^40^. However, our study found that the combination of Res and Gef synergistically induced Gef-resistant NSCLC cell death through triggering apoptosis, autophagy and senescence. These observations lead us to conclude that apoptosis is not sole determinant of the synergistic anticancer effects of Res and EGFR TKIs. Indeed, multiple cell death pathways are involved, and the complex interplay between them determines the therapeutic effects of combined Res and EGFR TKIs. Interestingly, we also revealed that Res inhibited Gef-related enzymes and transporters and modulated intracellular Gef pharmacokinetics.

Taken together, combined Gef and Res treatment overcomes Gef resistance in NSCLC cells by increasing the intracellular Gef concentration and inducing multiple cell death pathways. This study also provides an alternative perspective for studying the synergistic mechanisms of combination treatments with small molecular-targeted anticancer drugs, based on aspects of both intracellular drug pharmacokinetics and cell death processes.

## Methods

### Reagents

Gefitinib (Irresa) was purchased from AstraZeneca (Macclesfield, UK). The Annexin V-FITC Apoptosis Detection Kit, SA-β-gal Assay Kit, 4′,6-diamidino-2-phenylindole (DAPI) and Ac-DEVD-CHO (DEVD) were purchased from Beyotime (Shanghai, China). 3-methyladenine (3-MA), Resveratrol, monodansylcadaverine (MDC), and 3-(4,5-dimethyl-2-thiazolyl)-2,5-diphenyl-2H-tetrazolium bromide (MTT) were obtained from Sigma (St Louis, MO, USA). Primary antibodies against EGFR, phospho-EGFR (p^Y1068^-EGFR), p21^waf1/cip1^, p53, LC3B, caspase-3, beclin-1, and β-actin were obtained from Cell Signalling Technology (Beverly, MA, USA). Primary antibodies against CYP1A1, CYP2D6, ABCG2, and ABCG5 were obtained from Santa Cruz Biotechnology (Santa Cruz, CA).

### Cell lines and culture

The human NSCLC cell line PC9 was kindly provided by the Shanghai Pulmonary Disease Hospital affiliated with Tongji University. HCC827, A549, H1975, and H1299 cells were purchased from the cell bank of the Chinese Academy of Sciences (Shanghai, China). The Gef-resistant NSCLC cell lines PC9/G and HCC827/G were obtained as previously described^41^. The HCC827, HCC827/G, A549, H1975, and H1299 cell lines were cultured in RPMI 1640 medium. The PC9 and PC9/G cell lines were cultured in high-glucose DMEM medium, supplemented with 10% foetal bovine serum and 1% penicillin-streptomycin (Gibco, China), under a humidified atmosphere of 5% CO_2_ at 37 °C.

### Cell growth inhibition assay

The antiproliferative effects of the treatments were evaluated using the MTT assay. Cells were seeded at a density of 4 × 10^3^ cells/well in 96-well plates. After attachment, the culture media were replaced with various concentrations of Res and/or Gef for 72 h. Then, MTT assays were performed as described previously^42^.

Cells were seeded at a density of 4 × 10^3^ cells per well on 96-well plates, and after attachment, cells were treated with the following three combinations of drugs: (1) pretreated with Gef for 24 h followed by Res for 48 h (Gef → Res); (2) pretreated with Res for 24 h followed by Gef for 48 h (Res → Gef); and (3) treated concurrently with Gef and Res for 72 h (Gef + Res). The different drug doses were combined using constant ratios of the IC_50_ values calculated from the previous cytotoxicity tests. Then, we used 0.125, 0.25, 0.5, 1, 2, and 4 times the IC_50_ dose as the Gef and Res combination doses to calculate the CI value. The effects of different drug combinations were evaluated using the CompuSyn software (Biosoft, Ferguson, MO, USA) based on the median effect model of Chou and Talay^43^. The CI values were interpreted as follows: CI < 0.9, CI = 0.9–1.1, and CI > 1.1 indicated synergistic, additive and antagonistic effects, respectively.

### Determination of the intracellular accumulation of Gef

The accumulation studies in PC9 and PC9/G cells were initiated by adding DMEM medium containing various concentrations (1–20 μM) of Gef at 37 °C for 8 h. Cells were then washed in cold PBS, resuspended in water and subjected to three freeze-thaw cycles. Cells were then extracted with ethyl acetate containing internal standard (midazolam). Intracellular Gef concentrations were determined using liquid chromatography-tandem mass spectrometry (LC-MS/MS) as described by Alfieri *et al.*^5^. The analytes were ionized in positive ion mode, and the following MRM transitions were monitored: m/z 446.9 ([M + H]^+^) → 128.1 for Gef and m/z 326.0 ([M + H]^+^) → 291.0 for the internal standard (midazolam). For the intercellular pharmacokinetics of Gef in PC9/G cells, cells were exposed to Gef (1 μM) in the presence or absence of Res (40 μM). The dose selection was based on the fact that the maximum plasma concentration of Gef obtained at the clinically relevant dose (250 mg/day) is 1 μM^3^ and that the dose relevant to the possible biological effects of Res daily consumed from grape beverages is 40 μM^44^. Then, cell samples were collected at 0, 0.5, 1, 2.5, 5, 8 and 12 h. Intracellular Gef concentrations were determined using LC-MS/MS as described above and standardized to the total protein content of each sample. Kinetic analyses were carried out with the WinNonLin software (Pharsight, Mountain View, CA). Pharmacokinetics parameters (Ka, Ke, AUC, CL, T_max_, C_max_) of Gef were calculated with a one-compartmental open model.

### siRNA transfection

Three different siRNAs designed to knockdown CYP1A1 or ABCG2 and one pair of non-sense control RNAs were purchased from GenePharma (Shanghai, China). Transfection was performed using Opti-MEM medium and Lipofectamine 3000 (Invitrogen) according to the manufacturer’s protocol. The siRNA targeted sequences are listed in Supplementary Table S2. Cells (5 × 10^3^ cells/well) were cultured in 96-well plates. At 12 h after transfection, the cells were treated with different concentrations of Gef for 72 h, and then MTT assays were performed. Cells (4 × 10^5^ cells/well) were also seeded in 6-well plates. At 24 h after transfection, the cells were treated with 1 μM Gef for 8 h. Then, the intracellular concentrations of Gef were determined and the levels of EGFR and p-EGFR protein expression were detected.

### DAPI staining

Cells were seeded in 6-well plates at a density of 1 × 10^6^ cells/well. After attachment (24 h incubation), the medium was changed to 1% FBS in DMEM for a further 24 h to synchronize the cells. Then, the cells were divided into four groups: the control group (without drug intervention), the Res group (treated with 40 μM of Res), the Gef group (treated with 1 μM of Gef), and the Gef and Res group (treated with 40 μM of Res and 1 μM of Gef concomitantly). Cells were fixed with 4% paraformaldehyde and permeabilized with 0.3% Triton X-100. Cells were then stained with DAPI (1 mg/ml). Morphological changes in the nucleus were observed using a fluorescence microscope (Olympus IX70, Tokyo, Japan). Cells with condensed/fragmented and bright nuclei were considered apoptotic.

### Detection of cell apoptosis and cell cycle distribution by flow cytometry

For apoptosis analysis, cells were washed twice with 1× binding buffer then labelled with Annexin V and propidium iodide (PI) following the manufacturer’s instructions. The Apoptosis Analysis Kit was ordered from Tianjin Sungene Biotech (Tianjing, China). For cell cycle analysis, cells were washed twice with ice-cold PBS and fixed with 70% ethanol at −20 °C overnight. Then, the cells were washed with PBS and resuspended with a solution containing 50 μg/ml PI with 100 μg/ml RNase A in the dark at 37 °C for 30 min. The analysis of the samples was performed by flow cytometry (Becton-Dickinson, San Jose, CA, USA), and the acquired data were analysed with the CellQuest software (BD Biosciences).

### MDC staining

The autofluorescent substance MDC was used as a specific marker for autophagic vacuoles. Pretreated cells were collected and stained with MDC (50 μM) in the dark at 37 °C for 1 h as described by Biederbick *et al.*^45^. The cellular morphological changes were observed using a fluorescent microscope (Olympus, Tokyo, Japan) equipped with Motic Image Advanced 3.0 software. Cell fluorescence intensity was measured by flow cytometry to determine the autophagic ratio.

### SA-β-gal assay

Cell senescence was measured following the manufacturer’s protocol of the SA-β-gal assay kit. According to the protocol, cells were fixed with 2% paraformaldehyde for 15 min at room temperature and then incubated with fresh SA-β-gal stain solution in the dark at 37 °C overnight. SA-β-gal-positive cells were quantified by counting stained and unstained cells, and the result is presented as the ratio of SA-β-gal-positive cells over the total cells counted.

### Western blotting

Protein extraction, solubilization, and protein analysis by SDS-PAGE were performed as described elsewhere^42^. Briefly, equal amounts of protein (30–50 μg/lane) were resolved by SDS-PAGE and transferred onto PVDF membranes. The membranes were then incubated with each primary antibody, washed and subsequently incubated with horseradish peroxidase-conjugated secondary antibodies. Antibody binding was detected using an enhanced chemiluminescence system (Pierce Biotechnology, Rockford, IL). Quantitative analysis was carried out using a Gel-Pro Analyser (Media Cybernetics Inc.). The relative protein levels were quantified relative to an untreated control.

### Statistical Analysis

All data are expressed as means ± SD and analysed using two-tailed Student’s t-tests. Statistical analyses were performed using SPSS 18.0 (SPSS Inc., Chicago, IL, USA). A value of *P* < 0.05 was considered statistically significant.

## Additional Information

**How to cite this article**: Zhu, Y. *et al.* Resveratrol overcomes gefitinib resistance by increasing the intracellular gefitinib concentration and triggering apoptosis, autophagy and senescence in PC9/G NSCLC cells. *Sci. Rep.* **5**, 17730; doi: 10.1038/srep17730 (2015).

## Supplementary Material

Supplementary Information

## Figures and Tables

**Table 1:** Summary of intracellular Gef pharmacokinetic parameters in PC9/G cells treated with Gef (1 μM) alone or combined with Res (40 μM).

Parameters	Gef	Gef + Res
K_a_ (1/h)	0.30 ± 0.06	0.22 ± 0.04
K_e_ (1/h)	9.41 ± 1.61	7.41 ± 0.92
AUC (h*nM/mg protein)	441.3 ± 52.9	654.4 ± 57.3^*^
CL (mg protein /hr)	2.27 ± 0.27	1.27 ± 0.19^*^
T_max_ (h)	1.55 ± 0.18	1.17 ± 0.15
C_max_ (nM/mg protein)	28.45 ± 1.19	55.04 ± 1.95^**^

Data are presented as means ± SD (n = 3). *P < 0.05, **P < 0.01 compared with Gef treatment alone.
