# Supplementary material for: Resveratrol overcomes gefitinib resistance by increasing the intracellular gefitinib concentration and triggering apoptosis, autophagy and senescence in PC9/G NSCLC cells
Source: Sci Rep. 2015 Dec 4;5:17730. doi: 10.1038/srep17730 (PMC4669414; doi:10.1038/srep17730)
Supplement: Supplementary Information [file srep17730-s1.pdf]

Supplementary information for:

Resveratrol overcomes gefitinib resistance by increasing the intracellular gefitinib concentration and triggering apoptosis, autophagy and senescence in PC9/G NSCLC cells

Yinsong Zhu, Wenjuan He, Xiujuan Gao, Bin Li, Chenghan Mei, Rong Xu, Hui Chen

Supplementary Figure S1

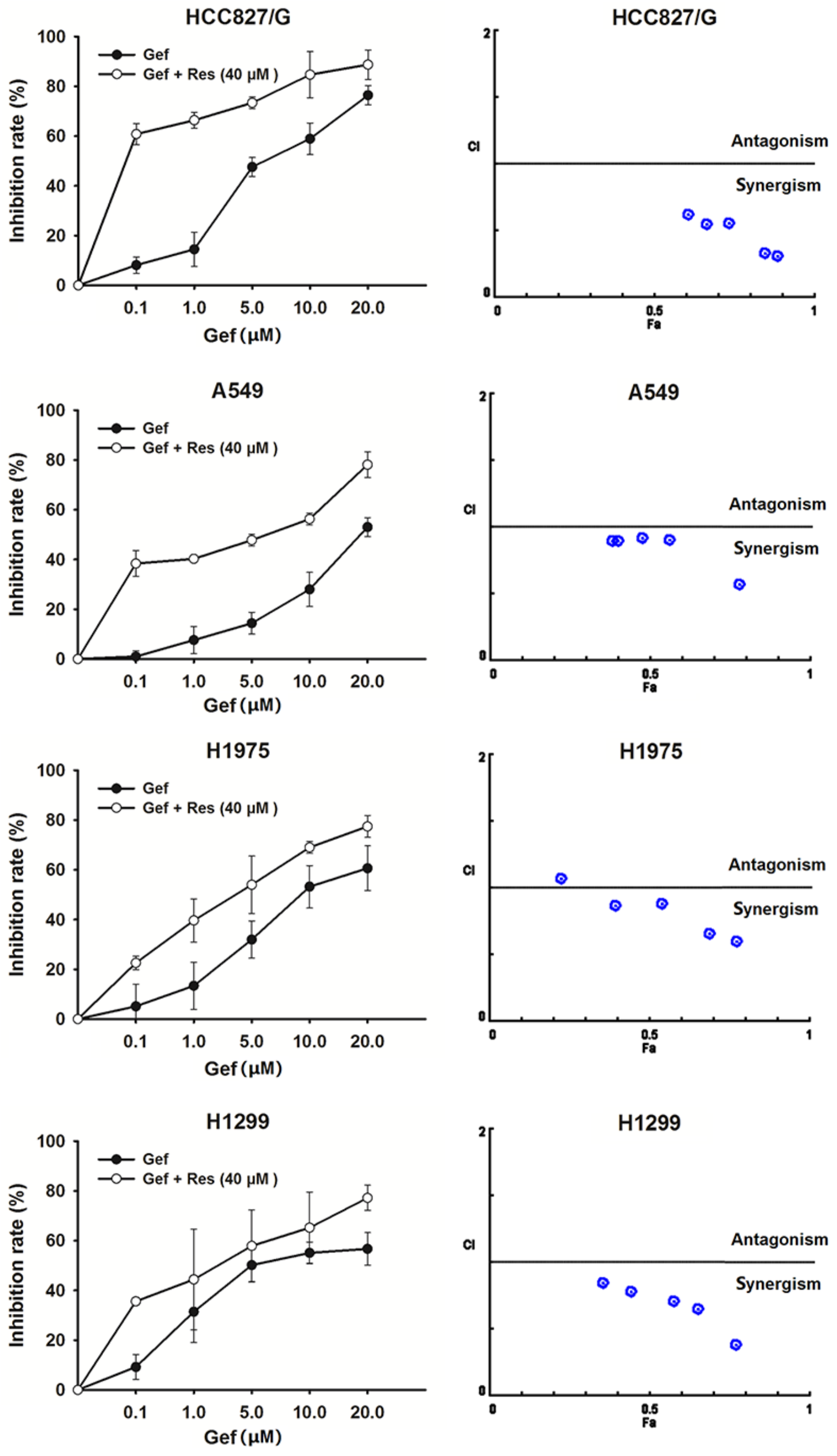

Supplementary Figure S1. The synergistic antiproliferative effects of Res and Gef in NSCLC cells. HCC827/G **(A)**, A549 **(B)**, H1975 **(C)**, and H1299 **(D)** cells were plated in 96-well plates and cultured in the presence of the indicated concentrations of Gef, alone or in combination with a fixed concentration of Res (40 μM) for 72 h. The viability of cells were assayed by MTT assay. Data are shown as means ± SD of three independent experiments. The CI values for Res and Gef in different cells were determined according to the Chou-Talalay's method. CI < 0.9, CI = 0.9 – 1.1, and CI > 1.1 indicated synergistic, additive and antagonistic effects, respectively.

Supplementary Table S1

Cell lines	EGFR mutation	K-Ras	Gef IC_{50} (μM)	Res IC_{50} (μM)
PC9	delE746-A750	WT	0.021 ± 0.005	82.50 ± 10.04
PC9/G	delE746-A750	WT	6.54 ± 0.58	33.90 ± 1.60
HCC827	delE746-A750	WT	0.014 ± 0.008	73.44 ± 4.32
HCC827/G	delE746-A750	WT	5.61 ± 1.48	42.94 ± 3.54
A549	WT	G12S	17.35 ± 3.96	72.39 ± 2.76
H1975	L858R/T790M	WT	11.52 ± 2.93	74.59 ± 2.03
H1299	WT	WT	7.08 ± 1.23	88.84 ± 1.62

Supplementary Table S1. The characteristics of NSCLC cell lines and the IC_{50} values of Gef and Res. The IC_{50} values for Gef > 1 μM was defined as Gef-resistant in NSCLC cell lines. Data are shown as means ± SD of three independent experiments.

Supplement Table S2

siRNA	Sequences
CYP1A1 siRNA-1	Sense: 5’-CCUUCACCCUCAUCAGUAATT-3’<br>Antisense: 5’-UUACUGAUGAGGGUGAAGGTT-3’
CYP1A1 siRNA-2	Sense: 5’-CCUAGUCAACCUGAAUAAUTT-3’<br>Antisense: 5’-AUUAUUCAGGUUGACUAGGTT-3’
CYP1A1 siRNA-3	Sense: 5’-GCUGGAUGAGAACGCCAAUTT-3’<br>Antisense: 5’-AUUGGCGUUCUCAUCCAGCTT-3’
ABCG2 siRNA-1	Sense: 5’-GGAGGCAAAUCUUCGUUAUTT-3’<br>Antisense: 5’-AUAACGAAGAUUUGCCUCCTT-3’
ABCG2 siRNA-2	Sense: 5’-GGCCUUGGGAUACUUUGAATT-3’<br>Antisense: 5’-UUCAAAGUAUCCCAAGGCCTT-3’
ABCG2 siRNA-3	Sense: 5’-GCCUACCUGAAAUUGUUAUTT-3’<br>Antisense: 5’-AUAACAAUUUCAGGUAGGCTT-3’
Control siRNA	Sense: 5’-UUCUCC- GAACGUGUCACGUTT-3’<br>Antisense: 5’-ACGUGACACGUUCGGAGAATT-3’

Supplement Table S2. Sequences of siRNAs targeting CYP1A1 and ABCG2.
